# Triphenyl­bis(2,4,5-trifluoro-3-methoxy­benzoato)anti­mony(V)

**DOI:** 10.1107/S1600536808029656

**Published:** 2008-09-20

**Authors:** Liyuan Wen, Handong Yin, Li Quan, Daqi Wang

**Affiliations:** a College of Chemistry and Chemical Engineering, Liaocheng University, Shandong 252059, People’s Republic of China

## Abstract

In the title compound, [Sb(C_6_H_5_)_3_(C_8_H_4_F_3_O_3_)_2_], the Sb atom lies on an inversion centre and exhibits a trigonal bipyramidal geometry with the axial positions occupied by the O atoms of two carboxyl­ate groups and the equatorial positions occupied by C atoms of the phenyl groups. Intra­molecular C—H⋯O hydrogen bonds stabilize the mol­ecular conformation. In the crystal structure, mol­ecules are connected by inter­molecular C—H⋯O hydrogen-bonding inter­actions, forming a layer structure parallel to (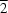
01).

## Related literature

For related structures, see: Ferguson *et al.* (1987 ▶); Ruether *et al.* (1985 ▶).
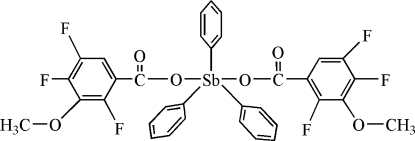


## Experimental

### 

#### Crystal data

[Sb(C_6_H_5_)_3_(C_8_H_4_F_3_O_3_)_2_]
*M* *_r_* = 763.27
Monoclinic, 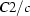

*a* = 12.7970 (14) Å
*b* = 22.890 (2) Å
*c* = 12.5131 (10) Å
β = 120.107 (2)°
*V* = 3170.9 (5) Å^3^
*Z* = 4
Mo *K*α radiation
μ = 0.95 mm^−1^
*T* = 293 (2) K
0.50 × 0.40 × 0.35 mm

#### Data collection

Bruker SMART area-detector diffractometer
Absorption correction: multi-scan (*SADABS*; Sheldrick, 1996 ▶) *T*_min_ = 0.635, *T*_max_ = 0.718
7869 measured reflections
2791 independent reflections
2338 reflections with *I* > 2σ(*I*)
*R*_int_ = 0.036

#### Refinement

*R*[*F* ^2^ > 2σ(*F* ^2^)] = 0.031
*wR*(*F* ^2^) = 0.092
*S* = 1.01
2791 reflections
215 parameters
H-atom parameters constrained
Δρ_max_ = 0.98 e Å^−3^
Δρ_min_ = −0.37 e Å^−3^

### 

Data collection: *SMART* (Siemens, 1996 ▶); cell refinement: *SAINT* (Siemens, 1996 ▶); data reduction: *SAINT*; program(s) used to solve structure: *SHELXS97* (Sheldrick, 2008 ▶); program(s) used to refine structure: *SHELXL97* (Sheldrick, 2008 ▶); molecular graphics: *SHELXTL* (Sheldrick, 2008 ▶); software used to prepare material for publication: *SHELXTL*.

## Supplementary Material

Crystal structure: contains datablocks I, global. DOI: 10.1107/S1600536808029656/rz2246sup1.cif

Structure factors: contains datablocks I. DOI: 10.1107/S1600536808029656/rz2246Isup2.hkl

Additional supplementary materials:  crystallographic information; 3D view; checkCIF report

## Figures and Tables

**Table 1:** Hydrogen-bond geometry (Å, °)

*D*—H⋯*A*	*D*—H	H⋯*A*	*D*⋯*A*	*D*—H⋯*A*
C10—H10⋯O2	0.93	2.36	3.053 (5)	131
C16—H16⋯O1	0.93	2.49	2.979 (5)	113
C8—H8*B*⋯O3^i^	0.96	2.57	3.240 (7)	127
C11—H11⋯O2^ii^	0.93	2.51	3.255 (5)	138

Symmetry codes: (i) 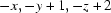; (ii) 
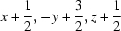
.

**Fig. 1.**
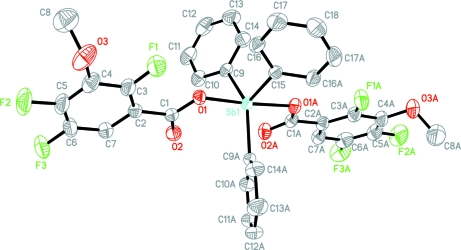
The molecular structure of the compound, showing 50% probability displacement ellipsoids. H atoms have been omitted for clarity. Symmetry code: (A) = 1 - x, y, 3/2 - z.

**Fig. 2.**
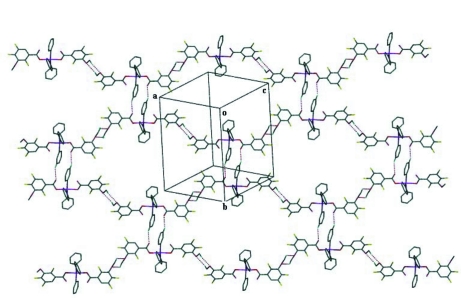
View of the two-dimensional layer structure in the title compound. Intermolecular hydrogen bonds are shown as dashed lines. H atoms are omitted.

## supplementary crystallographic information

### Comment

In recent years organoantimony(V) derivatives have attracted considerable attention due to their significant antimicrobial properties as well as antitumor activities. We have therefore synthesized the title compound, and present its crystal structure here.

The molecular structure of the compound is shown in Fig.1 The Sb atom, which lies on an inversion centre, assumes a distorted trigonal bipyramidal coordination geometry, provided by three phenyl groups at the equatorial positions and two carboxylate groups at the axial positions. The Sb—O bond lengths in organoantimony compounds are extremely variable, ranging from 1.935 Å in triphenylstibine oxide (Ferguson *et al.* 1987) to 2.506 Å in tetraphenylstibonium benzenesulphonate hydrate (Ruether *et al.* 1985). The Sb1—O1 distance (2.132 (2) Å) in the title compound lies within this range. The Sb—C bond distances (Sb1—C9 = 2.122 (3) Å; Sb1—C15 = 2.118 (5) Å) fall in the normal range for Sb—*C*(phenyl) bonds (2.10–2.13 Å). The molecular conformation is stabilized by C—H···O hydrogen bonds. In the crystal packing, molecules are linked by intermolecular C—H···O hydrogen bonds (Fig.2, Table 1,) into layers parallel to the (-2 0 1) plane.

### Experimental

The reaction was carried out under nitrogen atmosphere. 3-Methoxyl-2,4,5-trifluorobenzoic acid (2 mmol) and sodium ethoxide (2.4 mmol) were added to a stirred solution of methanol (30 ml) in a Schlenk flask and stirred for 0.5 h. Triphenylantimony dichloride (1 mmol) was then added to the reactor and the reaction mixture was stirred for 12 h at room temperature. The resulting clear solution was evaporated under vacuum. The product was crystallized from a dichloromethane/methanol (1:1 *v*/*v*) solution to yield colourless blocks of the title compound (yield 86%. m.p. 458 K). Anal. Calcd (%) for C_34_H_23_O_6_Sb_1_F_6_ (Mr = 763.27): C, 53.50; H, 3.04; F, 14.93; Sb, 15.95. Found (%): C, 53.55; H, 3.07; F, 14.89; Sb, 15.99

### Refinement

The H atoms were positioned geometrically, with methyl C—H distances of 0.96 Å and aromatic C—H distances of 0.93 Å, and refined as riding on their parent atoms, with *U*_iso_(H) = 1.2 *U*_eq_(C, O) or 1.5 *U*_eq_(C) for the methyl group.

### Crystal data

**Table:**

[Sb(C_6_H_5_)_3_(C_8_H_4_F_3_O_3_)_2_]	*F*(000) = 1520
*M**_r_* = 763.27	*D*_x_ = 1.599 Mg m^−^^3^
Monoclinic, *C*2/*c*	Mo *K*α radiation, λ = 0.71073 Å
Hall symbol: -C 2yc	Cell parameters from 3589 reflections
*a* = 12.7970 (14) Å	θ = 2.6–23.9°
*b* = 22.890 (2) Å	µ = 0.95 mm^−^^1^
*c* = 12.5131 (10) Å	*T* = 293 K
β = 120.107 (2)°	Block, colourless
*V* = 3170.9 (5) Å^3^	0.50 × 0.40 × 0.35 mm
*Z* = 4	

### Data collection

**Table:**

Bruker SMART area-detector diffractometer	2791 independent reflections
Radiation source: fine-focus sealed tube	2338 reflections with *I* > 2σ(*I*)
graphite	*R*_int_ = 0.036
φ and ω scans	θ_max_ = 25.0°, θ_min_ = 2.0°
Absorption correction: multi-scan (SADABS; Sheldrick, 1996)	*h* = −10→15
*T*_min_ = 0.635, *T*_max_ = 0.718	*k* = −27→23
7869 measured reflections	*l* = −14→14

### Refinement

**Table:**

Refinement on *F*^2^	Primary atom site location: structure-invariant direct methods
Least-squares matrix: full	Secondary atom site location: difference Fourier map
*R*[*F*^2^ > 2σ(*F*^2^)] = 0.031	Hydrogen site location: inferred from neighbouring sites
*wR*(*F*^2^) = 0.093	H-atom parameters constrained
*S* = 1.01	*w* = 1/[σ^2^(*F*_o_^2^) + (0.053*P*)^2^ + 2.3063*P*] where *P* = (*F*_o_^2^ + 2*F*_c_^2^)/3
2791 reflections	(Δ/σ)_max_ = 0.001
215 parameters	Δρ_max_ = 0.98 e Å^−^^3^
0 restraints	Δρ_min_ = −0.37 e Å^−^^3^

### Fractional atomic coordinates and isotropic or equivalent isotropic displacement parameters (Å^2^)

**Table:**

	*x*	*y*	*z*	*U*_iso_*/*U*_eq_	
Sb1	0.5000	0.601220 (13)	0.7500	0.04156 (14)	
F1	0.2452 (3)	0.54248 (11)	0.8929 (3)	0.0850 (8)	
F2	−0.0110 (3)	0.65440 (19)	0.9796 (3)	0.1229 (13)	
F3	0.0513 (3)	0.75229 (15)	0.9055 (3)	0.1104 (11)	
O1	0.3684 (2)	0.59621 (10)	0.8065 (2)	0.0501 (6)	
O2	0.3552 (2)	0.69304 (12)	0.7893 (3)	0.0614 (7)	
O3	0.0880 (4)	0.54671 (19)	0.9730 (4)	0.1077 (13)	
C1	0.3247 (3)	0.64637 (17)	0.8139 (3)	0.0501 (9)	
C2	0.2343 (3)	0.64523 (17)	0.8571 (3)	0.0515 (9)	
C3	0.2004 (4)	0.59594 (19)	0.8951 (4)	0.0608 (11)	
C4	0.1193 (4)	0.5976 (2)	0.9386 (5)	0.0745 (14)	
C5	0.0708 (4)	0.6507 (3)	0.9410 (5)	0.0808 (15)	
C6	0.1022 (4)	0.7004 (3)	0.9027 (5)	0.0789 (14)	
C7	0.1836 (3)	0.6985 (2)	0.8621 (4)	0.0612 (10)	
H7	0.2052	0.7327	0.8380	0.073*	
C8	0.1418 (6)	0.5380 (3)	1.1036 (6)	0.121 (2)	
H8A	0.2277	0.5425	1.1423	0.181*	
H8B	0.1233	0.4994	1.1192	0.181*	
H8C	0.1102	0.5663	1.1367	0.181*	
C9	0.6319 (3)	0.63273 (16)	0.9264 (3)	0.0455 (8)	
C10	0.6261 (4)	0.68703 (18)	0.9726 (4)	0.0609 (10)	
H10	0.5623	0.7123	0.9257	0.073*	
C11	0.7170 (4)	0.7030 (2)	1.0897 (4)	0.0752 (13)	
H11	0.7134	0.7390	1.1222	0.090*	
C12	0.8122 (5)	0.6661 (3)	1.1582 (4)	0.0837 (15)	
H12	0.8736	0.6775	1.2360	0.100*	
C13	0.8168 (4)	0.6123 (3)	1.1118 (5)	0.0858 (15)	
H13	0.8805	0.5870	1.1589	0.103*	
C14	0.7263 (4)	0.59551 (19)	0.9947 (4)	0.0675 (12)	
H14	0.7298	0.5593	0.9629	0.081*	
C15	0.5000	0.5087 (2)	0.7500	0.0441 (11)	
C16	0.4754 (4)	0.47799 (17)	0.8308 (4)	0.0601 (10)	
H16	0.4585	0.4981	0.8849	0.072*	
C17	0.4761 (5)	0.4175 (2)	0.8306 (5)	0.0791 (14)	
H17	0.4603	0.3972	0.8853	0.095*	
C18	0.5000	0.3873 (3)	0.7500	0.085 (2)	
H18	0.5000	0.3466	0.7500	0.102*	

### Atomic displacement parameters (Å^2^)

**Table:**

	*U*^11^	*U*^22^	*U*^33^	*U*^12^	*U*^13^	*U*^23^
Sb1	0.0500 (2)	0.0384 (2)	0.0411 (2)	0.000	0.02652 (16)	0.000
F1	0.107 (2)	0.0643 (16)	0.114 (2)	−0.0008 (14)	0.0786 (19)	0.0110 (15)
F2	0.092 (2)	0.195 (4)	0.124 (3)	0.005 (2)	0.086 (2)	−0.008 (3)
F3	0.105 (2)	0.126 (3)	0.121 (3)	0.0416 (19)	0.072 (2)	−0.008 (2)
O1	0.0571 (15)	0.0497 (15)	0.0566 (16)	0.0085 (11)	0.0384 (13)	0.0076 (12)
O2	0.0731 (17)	0.0541 (16)	0.0691 (19)	0.0055 (14)	0.0447 (15)	0.0113 (14)
O3	0.117 (3)	0.136 (3)	0.097 (3)	−0.050 (3)	0.074 (2)	−0.004 (3)
C1	0.048 (2)	0.061 (2)	0.042 (2)	0.0083 (17)	0.0229 (17)	0.0064 (17)
C2	0.0451 (19)	0.065 (2)	0.047 (2)	0.0059 (17)	0.0252 (17)	0.0075 (18)
C3	0.058 (2)	0.073 (3)	0.058 (3)	0.001 (2)	0.034 (2)	0.000 (2)
C4	0.064 (3)	0.105 (4)	0.066 (3)	−0.019 (3)	0.041 (2)	−0.001 (3)
C5	0.060 (3)	0.128 (5)	0.070 (3)	0.004 (3)	0.044 (2)	−0.005 (3)
C6	0.064 (3)	0.105 (4)	0.073 (3)	0.019 (3)	0.038 (2)	−0.010 (3)
C7	0.057 (2)	0.071 (3)	0.059 (2)	0.007 (2)	0.031 (2)	0.004 (2)
C8	0.121 (5)	0.135 (5)	0.110 (5)	−0.011 (4)	0.061 (4)	0.011 (5)
C9	0.052 (2)	0.047 (2)	0.041 (2)	−0.0099 (16)	0.0265 (16)	−0.0040 (16)
C10	0.072 (3)	0.055 (2)	0.060 (3)	−0.012 (2)	0.036 (2)	−0.010 (2)
C11	0.088 (3)	0.080 (3)	0.059 (3)	−0.033 (3)	0.039 (3)	−0.023 (2)
C12	0.077 (3)	0.116 (4)	0.051 (3)	−0.039 (3)	0.027 (2)	−0.027 (3)
C13	0.066 (3)	0.113 (4)	0.058 (3)	0.007 (3)	0.016 (2)	0.006 (3)
C14	0.062 (3)	0.077 (3)	0.052 (3)	0.003 (2)	0.020 (2)	−0.008 (2)
C15	0.048 (3)	0.043 (3)	0.040 (3)	0.000	0.022 (2)	0.000
C16	0.082 (3)	0.052 (2)	0.056 (2)	0.000 (2)	0.042 (2)	0.0034 (19)
C17	0.108 (4)	0.054 (3)	0.085 (4)	−0.005 (3)	0.056 (3)	0.013 (2)
C18	0.116 (6)	0.040 (3)	0.102 (6)	0.000	0.056 (5)	0.000

### Geometric parameters (Å, °)

**Table:**

Sb1—C15	2.118 (5)	C8—H8B	0.9600
Sb1—C9	2.122 (3)	C8—H8C	0.9600
Sb1—C9^i^	2.122 (3)	C9—C14	1.371 (5)
Sb1—O1^i^	2.132 (2)	C9—C10	1.388 (5)
Sb1—O1	2.132 (2)	C10—C11	1.387 (6)
F1—C3	1.358 (5)	C10—H10	0.9300
F2—C5	1.358 (5)	C11—C12	1.372 (7)
F3—C6	1.364 (6)	C11—H11	0.9300
O1—C1	1.301 (4)	C12—C13	1.376 (7)
O2—C1	1.228 (4)	C12—H12	0.9300
O3—C4	1.370 (6)	C13—C14	1.391 (6)
O3—C8	1.434 (7)	C13—H13	0.9300
C1—C2	1.504 (5)	C14—H14	0.9300
C2—C3	1.376 (5)	C15—C16^i^	1.390 (4)
C2—C7	1.398 (5)	C15—C16	1.390 (5)
C3—C4	1.393 (6)	C16—C17	1.384 (6)
C4—C5	1.371 (7)	C16—H16	0.9300
C5—C6	1.370 (8)	C17—C18	1.378 (6)
C6—C7	1.369 (6)	C17—H17	0.9300
C7—H7	0.9300	C18—C17^i^	1.378 (6)
C8—H8A	0.9600	C18—H18	0.9300
			
C15—Sb1—C9	109.87 (10)	H8A—C8—H8B	109.5
C15—Sb1—C9^i^	109.87 (10)	O3—C8—H8C	109.5
C9—Sb1—C9^i^	140.3 (2)	H8A—C8—H8C	109.5
C15—Sb1—O1^i^	86.91 (6)	H8B—C8—H8C	109.5
C9—Sb1—O1^i^	90.84 (12)	C14—C9—C10	120.7 (4)
C9^i^—Sb1—O1^i^	91.25 (12)	C14—C9—Sb1	115.4 (3)
C15—Sb1—O1	86.91 (6)	C10—C9—Sb1	123.8 (3)
C9—Sb1—O1	91.25 (12)	C11—C10—C9	118.9 (4)
C9^i^—Sb1—O1	90.84 (12)	C11—C10—H10	120.5
O1^i^—Sb1—O1	173.83 (12)	C9—C10—H10	120.5
C1—O1—Sb1	114.6 (2)	C12—C11—C10	120.7 (4)
C4—O3—C8	115.3 (5)	C12—C11—H11	119.7
O2—C1—O1	123.2 (3)	C10—C11—H11	119.7
O2—C1—C2	120.3 (3)	C11—C12—C13	119.9 (4)
O1—C1—C2	116.5 (3)	C11—C12—H12	120.0
C3—C2—C7	117.8 (3)	C13—C12—H12	120.0
C3—C2—C1	124.9 (4)	C12—C13—C14	120.2 (5)
C7—C2—C1	117.3 (3)	C12—C13—H13	119.9
F1—C3—C2	121.5 (3)	C14—C13—H13	119.9
F1—C3—C4	116.0 (4)	C9—C14—C13	119.5 (4)
C2—C3—C4	122.5 (4)	C9—C14—H14	120.2
O3—C4—C5	122.7 (5)	C13—C14—H14	120.2
O3—C4—C3	119.5 (5)	C16^i^—C15—C16	119.3 (5)
C5—C4—C3	117.8 (4)	C16^i^—C15—Sb1	120.3 (2)
F2—C5—C6	119.1 (5)	C16—C15—Sb1	120.3 (2)
F2—C5—C4	120.0 (5)	C17—C16—C15	120.0 (4)
C6—C5—C4	120.9 (4)	C17—C16—H16	120.0
F3—C6—C7	119.9 (5)	C15—C16—H16	120.0
F3—C6—C5	119.1 (4)	C18—C17—C16	120.5 (5)
C7—C6—C5	121.0 (5)	C18—C17—H17	119.7
C6—C7—C2	120.0 (4)	C16—C17—H17	119.7
C6—C7—H7	120.0	C17—C18—C17^i^	119.6 (6)
C2—C7—H7	120.0	C17—C18—H18	120.2
O3—C8—H8A	109.5	C17^i^—C18—H18	120.2
O3—C8—H8B	109.5		
			
C15—Sb1—O1—C1	−175.3 (2)	C1—C2—C7—C6	−178.9 (4)
C9—Sb1—O1—C1	74.9 (3)	C15—Sb1—C9—C14	25.4 (3)
C9^i^—Sb1—O1—C1	−65.5 (3)	C9^i^—Sb1—C9—C14	−154.6 (3)
Sb1—O1—C1—O2	0.8 (5)	O1^i^—Sb1—C9—C14	−61.7 (3)
Sb1—O1—C1—C2	−178.2 (2)	O1—Sb1—C9—C14	112.5 (3)
O2—C1—C2—C3	−175.3 (4)	C15—Sb1—C9—C10	−154.6 (3)
O1—C1—C2—C3	3.8 (6)	C9^i^—Sb1—C9—C10	25.4 (3)
O2—C1—C2—C7	2.9 (5)	O1^i^—Sb1—C9—C10	118.3 (3)
O1—C1—C2—C7	−178.1 (3)	O1—Sb1—C9—C10	−67.5 (3)
C7—C2—C3—F1	179.9 (4)	C14—C9—C10—C11	−0.5 (6)
C1—C2—C3—F1	−2.0 (6)	Sb1—C9—C10—C11	179.5 (3)
C7—C2—C3—C4	−0.5 (6)	C9—C10—C11—C12	1.0 (7)
C1—C2—C3—C4	177.6 (4)	C10—C11—C12—C13	−1.4 (7)
C8—O3—C4—C5	−77.2 (7)	C11—C12—C13—C14	1.2 (8)
C8—O3—C4—C3	105.1 (6)	C10—C9—C14—C13	0.4 (7)
F1—C3—C4—O3	−1.5 (7)	Sb1—C9—C14—C13	−179.6 (4)
C2—C3—C4—O3	178.9 (4)	C12—C13—C14—C9	−0.7 (8)
F1—C3—C4—C5	−179.3 (4)	C9—Sb1—C15—C16^i^	−119.9 (2)
C2—C3—C4—C5	1.1 (7)	C9^i^—Sb1—C15—C16^i^	60.1 (2)
O3—C4—C5—F2	0.6 (8)	O1^i^—Sb1—C15—C16^i^	−30.1 (2)
C3—C4—C5—F2	178.3 (4)	O1—Sb1—C15—C16^i^	149.9 (2)
O3—C4—C5—C6	−178.2 (5)	C9—Sb1—C15—C16	60.1 (2)
C3—C4—C5—C6	−0.5 (8)	C9^i^—Sb1—C15—C16	−119.9 (2)
F2—C5—C6—F3	0.6 (7)	O1^i^—Sb1—C15—C16	149.9 (2)
C4—C5—C6—F3	179.5 (5)	O1—Sb1—C15—C16	−30.1 (2)
F2—C5—C6—C7	−179.5 (4)	C16^i^—C15—C16—C17	0.3 (3)
C4—C5—C6—C7	−0.7 (8)	Sb1—C15—C16—C17	−179.7 (3)
F3—C6—C7—C2	−178.9 (4)	C15—C16—C17—C18	−0.6 (7)
C5—C6—C7—C2	1.3 (7)	C16—C17—C18—C17^i^	0.3 (3)
C3—C2—C7—C6	−0.7 (6)		

Symmetry codes: (i) −*x*+1, *y*, −*z*+3/2.

### Hydrogen-bond geometry (Å, °)

**Table:**

*D*—H···*A*	*D*—H	H···*A*	*D*···*A*	*D*—H···*A*
C10—H10···O2	0.93	2.36	3.053 (5)	131
C16—H16···O1	0.93	2.49	2.979 (5)	113
C8—H8B···O3^ii^	0.96	2.57	3.240 (7)	127
C11—H11···O2^iii^	0.93	2.51	3.255 (5)	138

Symmetry codes: (ii) −*x*, −*y*+1, −*z*+2; (iii) *x*+1/2, −*y*+3/2, *z*+1/2.
